# Retrospective analysis of OCT on MB characteristics and 1-year follow-up of the ISR incidence after the DES implantation in patients with MB

**DOI:** 10.1038/s41598-021-04579-9

**Published:** 2022-01-11

**Authors:** Tian Xu, Wei You, Zhiming Wu, Peina Meng, Fei Ye, Xiangqi Wu, Shaoliang Chen

**Affiliations:** Department of Cardiology, Nanjing First Hospital, Nanjing Medical University, 68 Changle Rd, Nanjing, 210006 China

**Keywords:** Cardiology, Interventional cardiology

## Abstract

We used optical coherence tomography (OCT) to analyze the "half-moon" like phenomenon and its characteristics and observe 1-year follow-up of the in-stent restenosis (ISR) incidence after the drug eluted stent (DES) implantation in patients with the myocardial bridge (MB). Patients were retrospectively analyzed from January 2013 to December 2019. We used OCT to check 45 patients with MB and found a visible muscle layer (VML) around the vessel adventitia with the same or high density compared to the vessel media layer. There was not any significant difference in maximal thickness, maximal arch, and total length between the half-moon layer and the visible muscle layer groups (*p* > 0.05). Maximal thickness, arch, and total length of the half-moon layer were significantly positively related to VML, respectively (*r* = 0.962, 0.985, 0.742, *p* < 0.01). Of these 626 patients with MB seen by OCT, only 300 could be checked out by coronary angiography (CAG). Besides, the larger the thickness and arch of the VML around the vessel adventitia, the more severe the MB in these patients (*p* < 0.05). After the OCT use, there was no coronary perforation in these patients with MB covered with DES. After 1-year follow-up, ISR in MB covered with DES showed a notable difference among no MB, mild MB, moderate MB, and severe MB groups (*p* < 0.05), and ISR in DES aggravated with the MB severity. However, ISR in MB with and without covered with DES had no significant difference among the 4 groups (*p* > 0.05). OCT could evaluate MB characteristics accurately compared to IVUS and had a higher rate of detecting MB than CAG. Moreover, it is safe and effective to guide DES covering the mild MB segment in patients with severe coronary lesions detected by the OCT.

## Introduction

The heart is supplied by the branches of the left and right coronary arteries^1^. Because these vessels and their main branches are distributed along the heart surface, they are called epicardial coronary arteries^2^. However, some coronary arteries may be embedded in the myocardium, and the myocardial bridge (MB) is a myocardial bundle through which coronary segment tunnels and could compress coronary arteries causing myocardial ischemia^3^. MB is a congenital coronary variant, mainly affecting the left anterior descending coronary artery (LAD)^4^. The most extensive autopsy report included 1056 subjects, with an MB prevalence of 26%, of which 88% involved the LAD^5^. A population-based computed tomography (CT) study estimated the MB prevalence to be 22.5%^6^. Therefore, an estimated MB prevalence of about 25% is generally accepted. Most patients with MB have no symptoms, but a small number of patients develop angina, which may be secondary to the dynamic myocardial ischemia of the septal branch in the bridge segment^7^. Furthermore, symptoms may develop or progress with age, which is caused by diastolic heart dysfunction, endothelial vessel dysfunction and left ventricular hypertrophy, etc.^7^. Atherosclerotic plaque may accumulate proximal to the MB, which may be due to the dynamic retrograde flow caused by the bridge segment compression^8^. Besides, various clinical syndromes were reported to be associated with the MB, including coronary artery spasm, acute coronary syndrome, ventricular arrhythmia, and even sudden cardiac death^7,9^.

There are several methods for the diagnosis of MB, including noninvasive and invasive types^9^. The noninvasive modality for diagnosing MB is cardiac CT, which can easily evaluate the length and thickness of MB in the coronary artery^9,10^. The invasive techniques for diagnosing MB are coronary angiography (CAG) and IVUS (intravascular ultrasound)^9,11^. A significant "milking effect" is present when minimal luminal diameter (MLD) decreases by ≥ 70% during systole and ≥ 35% during mid-to-late diastole^12^. Using IVUS, the characteristic finding is a "half-moon" sign. Only there is an echolucent area between the MB segment and epicardial tissue, which lasts for the whole cardiac cycle^11^. Optical coherence tomography (OCT) is a high-resolution (10–20 μm) intravascular imaging technique that uses near-infrared light. Bose et al. have first reported a male case with chest pain that OCT showed no evidence of atherosclerosis but did show that the vessels were patent in diastole and collapsed in systole^13^. Liu and his colleagues have documented that OCT detected a sharp border and heterogeneous, signal-poor fusiform area indicative of arterial tunneling through the myocardium different from the echolucent muscle layer shown on IVUS in patients with MB^14^. Recently, Liu and his team have revealed that MB is both smaller and thinner than that of the adjacent non-MB segment, explaining why the increased frequency and severity of coronary artery perforation during the stent implantation in the MB segment^15^. However, no data to date reported what the "half-moon" like area shown by the IVUS corresponds to under the OCT detection.

Therefore, in the present study, we first found that the visible muscle layer around the coronary artery adventitia using OCT corresponded to the "half-moon" area using IVUS and then tested the relation between their characteristics in 45 patients with MB detected by both IVUS and OCT. Next, we analyzed the visible muscle layer characteristics in detail according to the MB grading after the CAG evaluation. Finally, we retrospectively studied the clinical significance of OCT for guiding the DES implantation in patients with MB.

## Methods

### Study design

**Figure 1:**
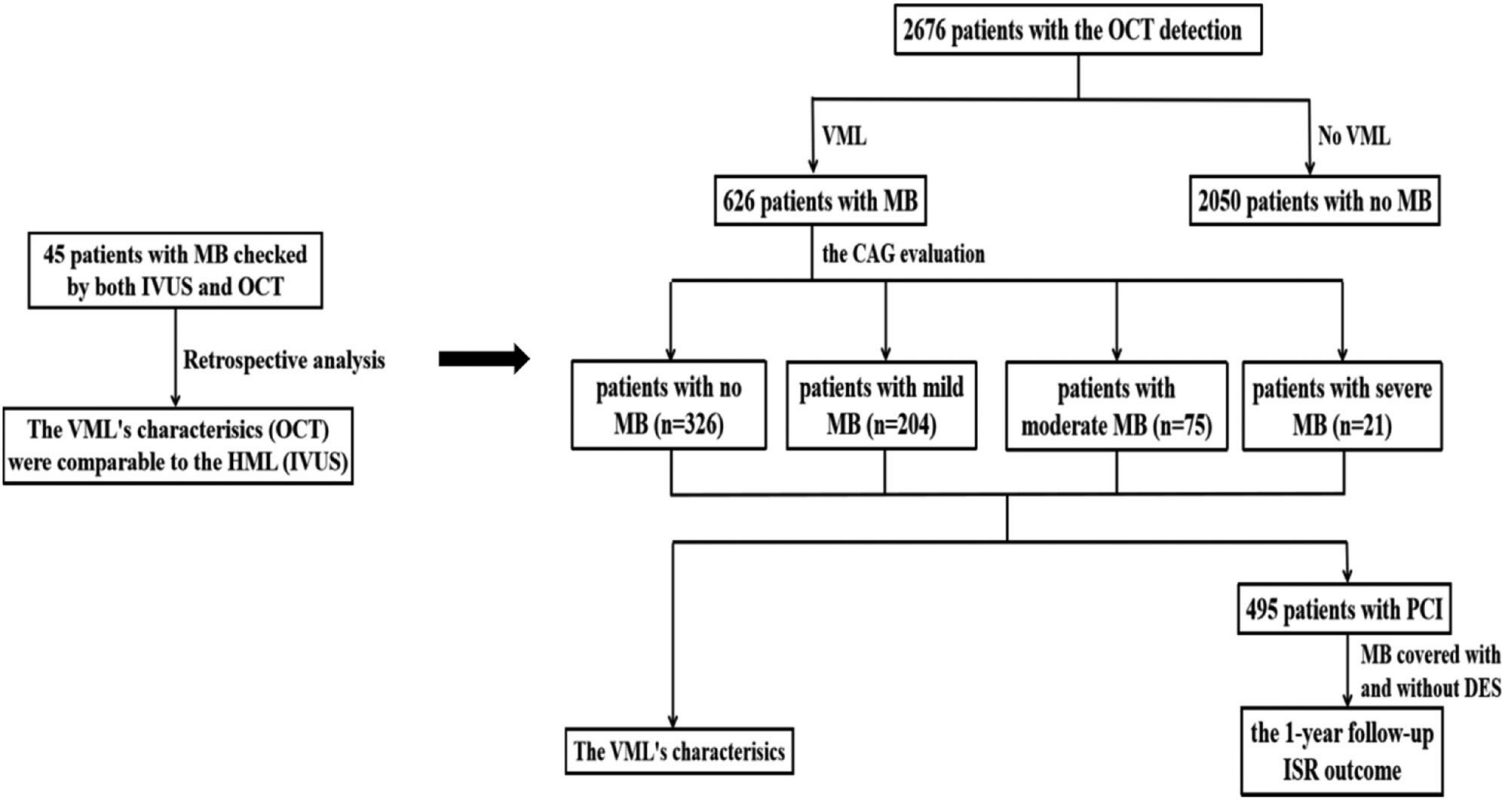
Work flow chart. *MB* myocardial bridge, *IVUS* intravascular ultrasound, *OCT* optical coherence tomography, *VML* visible muscle layer, *HML* half-moon layer, *CAG* coronary angiography, *PCI* percutaneous coronary intervention, *ISR* in-sent restenosis.

It was a retrospective study. First, a total of 45 patients with MB received both IVUS and OCT detection. According to the CAG grading ^16^, these patients were divided into no MB (n = 25), mild MB (n = 16), and moderate to severe MB (n = 4) groups. Second, 2676 patients were split into no MB (n = 2050) and MB (n = 626) groups after the OCT checking. Third, we further divided 626 patients with MB into no MB (n = 326), mild MB (n = 204), moderate MB (n = 75), and severe MB (n = 21) groups after the CAG evaluation again. Finally, after a 1-year follow-up, the incidence of in-stent restenosis (ISR) was analyzed in these patients. The workflow chart of this study was seen in Fig. 1. All the above-mentioned patients were enrolled from January 2013 to December 2019 in our hospital. The ethics committee of Nanjing First Hospital approved the study protocol, and all methods were performed in accordance with relevant guidelines and regulations. Additionally, written informed consent was obtained from all patients before cardiac catheterization.

### CAG, percutaneous coronary intervention (PCI), and quantitative coronary angiography (QCA) procedures^17^

Standard techniques performed CAG. Angiographic images were obtained after intracoronary injection of nitrate (100 or 200 mg). All angiograms were analyzed at a core laboratory (Nanjing First Hospital, Nanjing, China) in a blinded fashion. QCA was performed in optimal projections with a validated automated edge-detection coronary system (CAAS version 5.7, Pie Medical Imaging, Netherlands). MLD, reference vessel diameter (RVD), and percent diameter stenosis (%DS) were measured. According to the report of Noble et al., the degree of narrowing of the LAD during systole was graded 3 (greater than 75 percent), graded 2 (50 to 75 percent), and graded 1 (less than 50 percent)^16^.

All procedures were performed according to the current PCI guideline. During the procedure, unfractionated heparin was used to maintain activated coagulation time > 250 s. If the procedure time were more than 1 h, an additional 3000 IU of heparin would be added. If aspirin and clopidogrel were not used before admission, it was recommended that all patients took a loading dose of aspirin (300 mg) and clopidogrel (600 mg, or ticagrelor 180 mg) at least 2 h before PCI. The selection of DES type, procedure technique, and the use of glycoprotein IIb/IIIa inhibitors was at the operator's discretion. The immediate criteria for the successful PCI procedure were that the stenosis lumen was significantly enlarged after the DES implantation or the drug-coated balloon (DCB) therapy. The residual DS was less than 20%, and TIMI blood flow was grade 3. After PCI, all patients were prescribed aspirin 100 mg daily and clopidogrel 75 mg daily (ticagrelor 90 mg twice daily) for at least 12 months.

### IVUS and OCT procedures^18,19^

**Figure 2:**
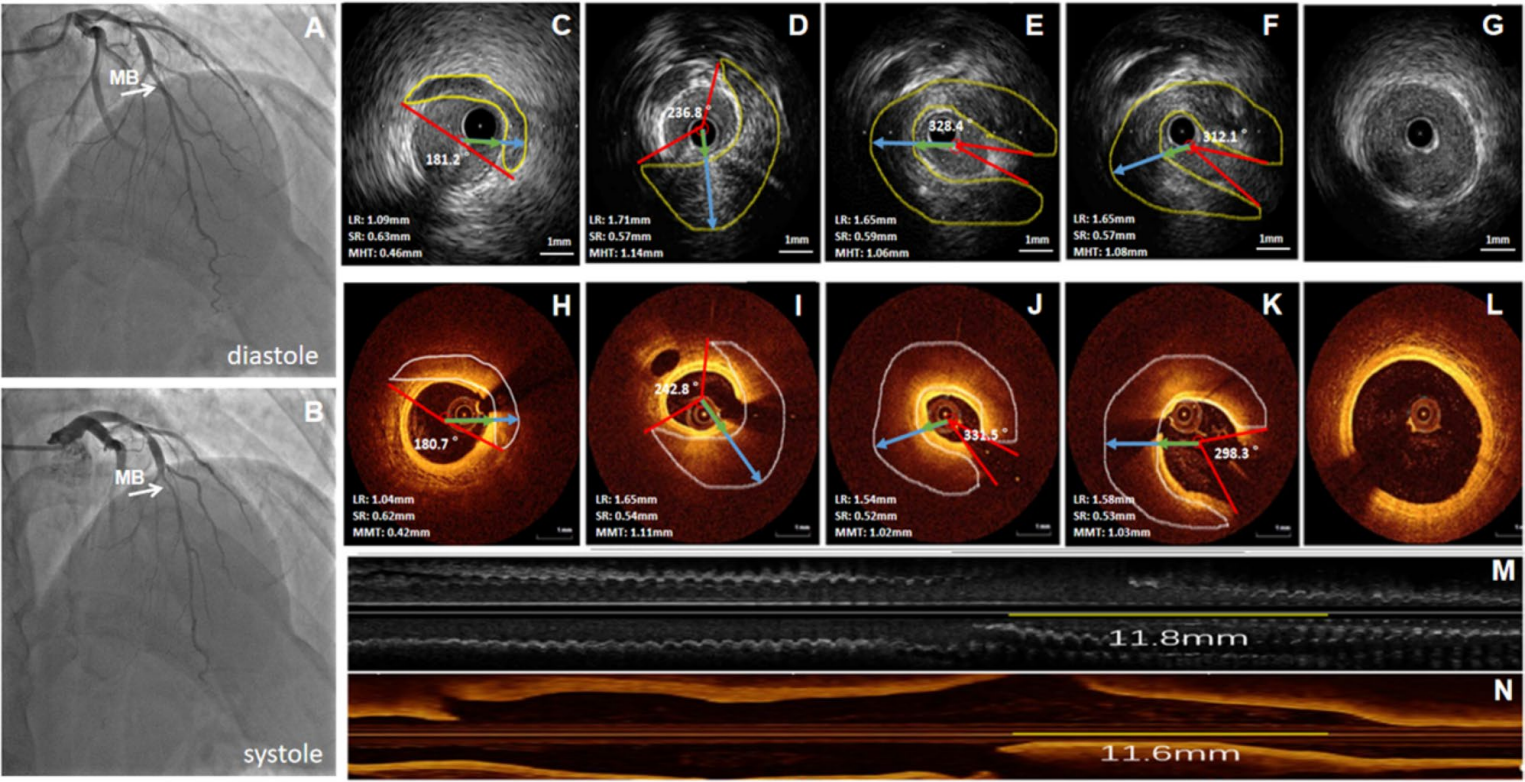
The representative case with MB was shown to measure indexes about the "half-moon" area and the muscle layer around the vessel's adventitia. The male patient with MB was 53 years old, and LAD was checked with CAG, IVUS, and OCT. (**A**,**B**) CAG results of MB. MB was located in the middle segment of LAD. The MLD in the MB segment was 2.43 mm during diastole and 0.97 mm during systole, and the compression rate of MB was 60%. (**C**–**G**,**M**) IVUS results of MB. (**C**) Showed the MHT was 0.46 mm, and the HA was 181.2°. (**D**) Showed the MHT was 1.14 mm, and the HA was 236.8°. (**E**) Showed the MHT was 1.06 mm, and the HA was 328.4°. (**F**) Showed the MHT was 1.08 mm, and the HA was 312.1°. (**G**) Showed there was no "half-moon" area in the proximal cross-section. (**M**) Showed the total length of the "half-moon" area was 11.8 mm. (**H**–**L**,**N**) OCT results of MB. (**H**) Showed the MMT was 0.42 mm, and the MA was 180.7°. (**I**) Showed the MMT was 1.11 mm, and the MA was 242.8°. (**J**) Showed the MMT was 1.02 mm, and the MA was 331.5°. (**K**) Showed the MMT was 1.03 mm, and the MA was 298.3°. (**L**) Showed there was no muscle layer in the proximal cross-section. (**N**) Showed the total length of the muscle layer was 11.6 mm. Scale bar: 1 mm. *MB* myocardial bridge, *LAD* left anterior descending artery, *CAG* coronary angiography, *IVUS* intravascular ultrasound, *OCT* optical coherence tomography, *MLD* minimum lumen diameter, *LR* long radius, *SR* short radius, *MHT* maximal "half-moon" layer’s thickness, *HA* "half-moon" layer’s arch, *MMT* maximal muscle layer’s thickness, *MA* muscle layer’s arch.

After intracoronary injection of nitroglycerin (100–200 mg), the IVUS catheter was pushed at least 10 mm distal to the lesion or stent edge. IVUS images were obtained through the automatic pullback (0.5 mm/s) by a commercially available imaging system with a 40-MHz mechanical transducer (Boston Scientific, Natick, Massachusetts) for measuring on-site. All IVUS images were stored on a DVD for offline measurements. A "half-moon" like echolucent area surrounding the coronary artery was seen during the whole cardiac diastole and systole cycle. Mark the range of a “half-moon” layer (HML) manually on the cross-section, and the HML’s thickness was the difference between long and short radii. Next, choose out the maximal HML thickness. Then, take 4 cross-sections of the HML on average and calculate the mean value of the maximal HML’s thickness of these 4 cross-sections for a patient. First, take the vessel center point as the vertex from the cross-section, and the two ends of HML were points A and B respectively. Second, the angle between points A and B was the HML’s arch. Finally, take an average of 4 cross-sections to find out the maximal HML’s arch for a patient. Find out the starting point and endpoint of HML on both the cross-section and the long axis plane, and calculate the length of HML on the long axis plane. If there is discontinuity, calculate the sum of the lengths of each HML part. The representative case with MB was shown how to measure indexes about the "half-moon" area (Fig. 2).

**Figure 3:**
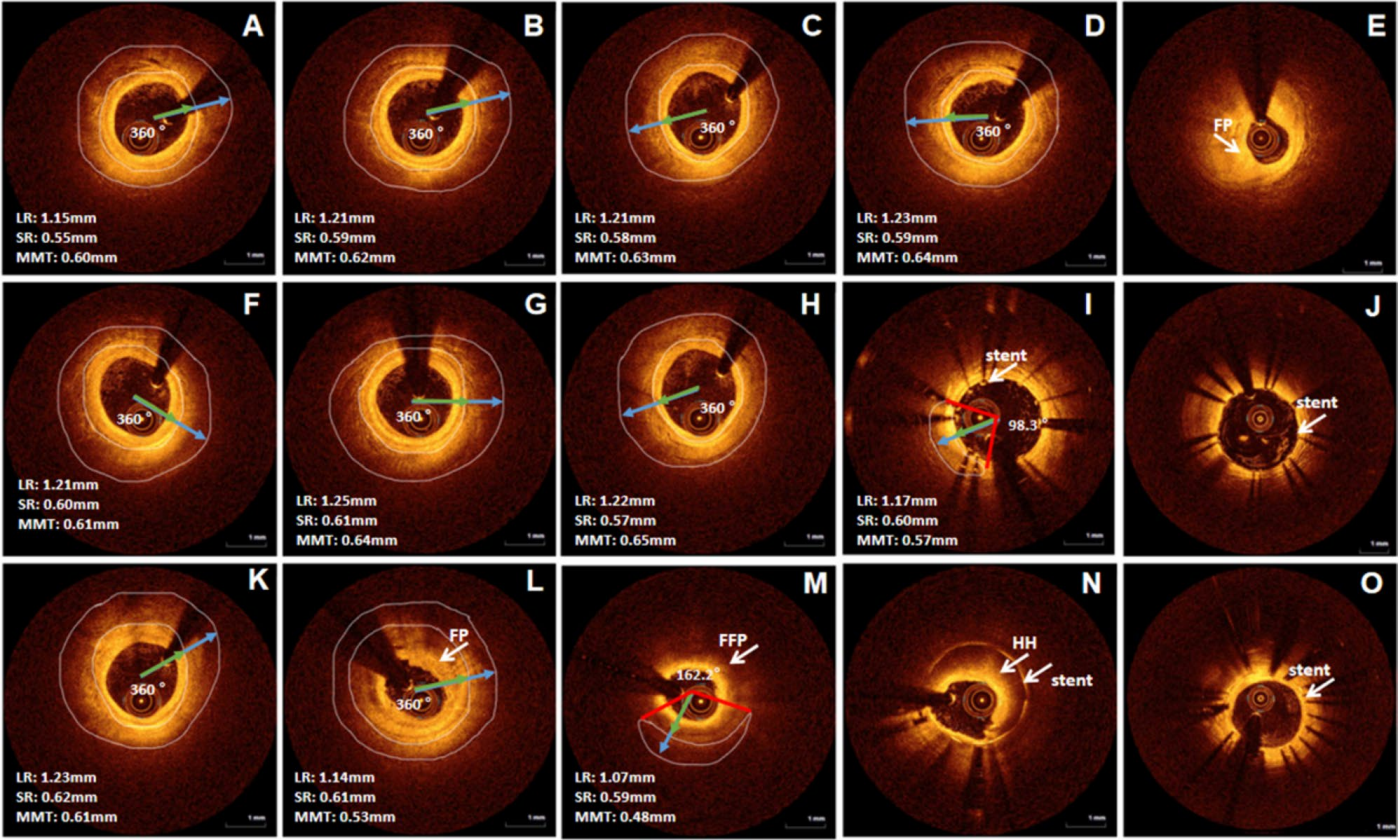
The representative case was about the dynamic observation of the MB change detected by OCT through 1-year follow-up. The male patient with MB was 57 years old, and two DESs (3.5 × 36 mm, 3.5 × 12 mm) were implanted in proximal and middle LAD. (**A**–**E**) Before PCI. (**A**) Showed the MMT was 0.60 mm, and the MA was 360°; (**B**) showed the MMT was 0.62 mm, and the MA was 360°; (**C**) showed the MMT was 0.63 mm, and the MA was 360°; (**D**) showed the MMT was 0.64 mm, and the MA was 360°; (**E**) showed there was FP in the middle LAD. (**F**–**J**) Immediately post PCI; (**F**) showed the MMT was 0.61 mm, and the MA was 360°; (**G**) showed the MMT was 0.64 mm, and the MA was 360°; (**H**) showed the MMT was 0.65 mm, and the MA was 360°; (**I**) showed the MMT was 0.57 mm, the MA was 98.3°, and the implanted coronary stent significantly reduced the MB size; (**J**) showed there was coronary stent in the middle LAD. (**K**–**O**) 1-year follow-up post PCI; (**K**) showed the MMT was 0.61 mm, and the MA was 360°; (**L**) showed the MMT was 0.53 mm, the MA was 360°, and FP slightly influenced the MB size; (**M**) showed the MMT was 0.48 mm, the MA was 162.2°, and FFP significantly decrease the MB size; (**N**) showed MB disappeared because stent implantation and HH; (**O**) showed the good endothelialization of coronary stents. Scale bar: 1 mm. *DES* drug eluted stent, *FP* fibrous plaque, *FFP* fibrous-fatty plaque, *HH* heterogeneous hyperplasia.

After an intracoronary administration of 1.0 mg nitroglycerin, OCT imaging of the main vessel was serially performed three times (before the intervention, immediately post-intervention, 12 months post-intervention) in all patients using a frequency-domain OCT system (LightLab Imaging, Inc., St. Jude Medical, St. Paul, MN, USA). In this study, an OCT catheter was advanced for imaging at least 10 mm distal to the target lesion. Generation of OCT cross-sectional images was at a rate of 100 frames/s while the fiber optic probe was withdrawn within the stationary protective sheath at a speed of 20 mm/s. All OCT images were analyzed by proprietary offline software (LightLab Imaging or St. Jude Medical) by one independent investigator who was blinded to patient and procedural information. A visible muscle layer (its homogeneous density was greater than or equal to that of the middle layer) around the vessel adventitia was also seen during the whole cardiac cycle. Mark the range of a visible muscle layer (VML) manually on the cross-section, and the VML’s thickness was the difference between long and short radii. Next, choose out the maximal VML thickness. Then, take 4 cross-sections of the VML on average and calculate the mean value of the maximal HML’s thickness of these 4 cross-sections for a patient. First, take the vessel center point as the vertex from the cross-section, and the two ends of VML were points A and B, respectively. Second, the angle between points A and B was the VML’s arch. Finally, take an average of 4 cross-sections to find out the maximal HML’s arch for a patient. Find out the starting point and endpoint of VML on both the cross-section and the long axis plane, and calculate the length of VML on the long axis plane. If there is discontinuity, calculate the sum of the lengths of each VML part. The representative case with MB was shown how to measure indexes about the muscle layer (Fig. 2). Furthermore, we supplied the representative case about the dynamic observation of the MB change detected by OCT through a 1-year follow-up (Fig. 3). The fibrous plaque has high backscattering and a relatively homogeneous OCT signal. Calcified plaque is defined as a plaque with a delineated, signal-poor region with low backscatter. Lipid plaque refers to the plaque with a diffusely bordered, signal-poor region with rapid signal attenuation^20^.

### Follow-up procedure and the primary endpoint

We routinely performed clinical follow-up at 1, 6, 9, and 12 months after the DES implantation, and CAG at the same time at the 12th month in our hospital. ISR was the primary endpoint of our retrospective study after 1-year follow-up. It was defined as stent diameter restenosis greater than 50% in CAG^18^.

### Statistical analysis

The Kolmogorov–Smirnov test assessed the distribution of continuous variables. Continuous variables were expressed as mean ± SD for normal distribution and compared using the Student's t-test or described as the median for non-normal distribution and compared using the Mann–Whitney U test. Categorical variables were expressed as frequencies or percentages and compared by chi-square statistics or the Fisher exact test. A *p*-value < 0.05 was considered statistically significant. All analyses were performed using the statistical program SPSS24.0 (SPSS Institute, Chicago, Illinois).

### Ethical approval

The ethics committee of Nanjing First Hospital approved the study protocol, and all methods were performed in accordance with relevant guidelines and regulations. Additionally, written informed consent was obtained from all patients before cardiac catheterization.

## Results

### IVUS and OCT characteristics about MB among no MB, mild MB, and moderate to severe MB groups according to CAG

45 patients with MB were detected by the IVUS and divided into no MB, mild MB, and moderate to severe MB groups according to the CAG standard for the MB classification. There was not any significant difference in maximal thickness, arch, and total length of the half-moon layer among the three groups (*p* > 0.05). However, these 3 indexes had a significant increase tendency in the moderate to severe MB group (Table 1).

**Table 1:** IVUS and OCT characteristics about MB among no MB, mild MB, and moderate to severe MB groups according to coronary angiography.

Variables	No MB (n = 25)	Mild MB (n = 16)	Moderate to severe MB (n = 4)	*P*1 value	*P*2 value	*P*3 value	*P*4 value
**IVUS**							
The maximal half-moon layer’s thickness	0.71 ± 0.20	0.72 ± 0.19	0.85 ± 0.21	0.472	0.852	0.225	0.287
The maximal half-moon layer’s arch	252.58 ± 94.01	272.78 ± 95.41	316.80 ± 74.82	0.439	0.516	0.222	0.418
Total half-moon layer’s length	15.18 ± 7.03	16.66 ± 7.88	22.91 ± 6.10	0.172	0.541	0.063	0.145
**OCT**							
The maximal visible muscle layer’s thickness	0.70 ± 0.22	0.73 ± 0.20	0.78 ± 0.11	0.756	0.652	0.497	0.691
The maximal visible muscle layer’s arch	245.22 ± 97.98	271.56 ± 96.55	314.85 ± 78.20	0.378	0.412	0.200	0.440
Total visible muscle layer’s length	11.36 ± 6.13	13.97 ± 6.41	21.10 ± 8.98	0.032	0.235	0.011	0.066

Data were expressed as n (%), mean ± SD. Variance analysis, P1; Mild MB vs. No MB, P2; Moderate or severe MB vs. No MB, P3; Moderate or severe MB vs Mild MB, P4.

*IVUS* intravascular ultrasound, *OCT* optical coherence tomography, *MB* myocardial bridge.

Meanwhile, we also used the OCT to check these 45 patients with MB. We found a visible muscle layer around the vessel adventitia with the same or high density as the vessel media layer. The muscle layer's location detected by the OCT was comparable as the IVUS shows the half-moon layer. Then, maximal thickness, arch, and total length of the muscle layer were measured. These 3 indexes among no MB, mild MB and moderate to severe MB groups showed no significant difference (*p* > 0.05), but they also had a notable increase tendency in the moderate to severe MB group (Table 1). Next, respective characteristics of the visual signs about MB detected by IVUS and OCT were shown in Table 2. We found that there was not any significant difference in maximal thickness, maximal arch, and total length between the half-moon layer and the visible muscle layer groups (*p* > 0.05).

**Table 2:** Respective characteristics of the visual signs about MB detected by IVUS and OCT.

	The half-moon layer (IVUS, n = 45)	The visible muscle layer (OCT, n = 45)	*p* value
The maximal thickness (mm)	0.73 ± 0.20	0.72 ± 0.21	0.827
The maximal arch (°)	265.47 ± 94.81	260.78 ± 98.12	0.820
Total length (mm)	16.39 ± 7.58	13.15 ± 7.09	0.052

Data were expressed as mean ± SD.

**Figure 4:**
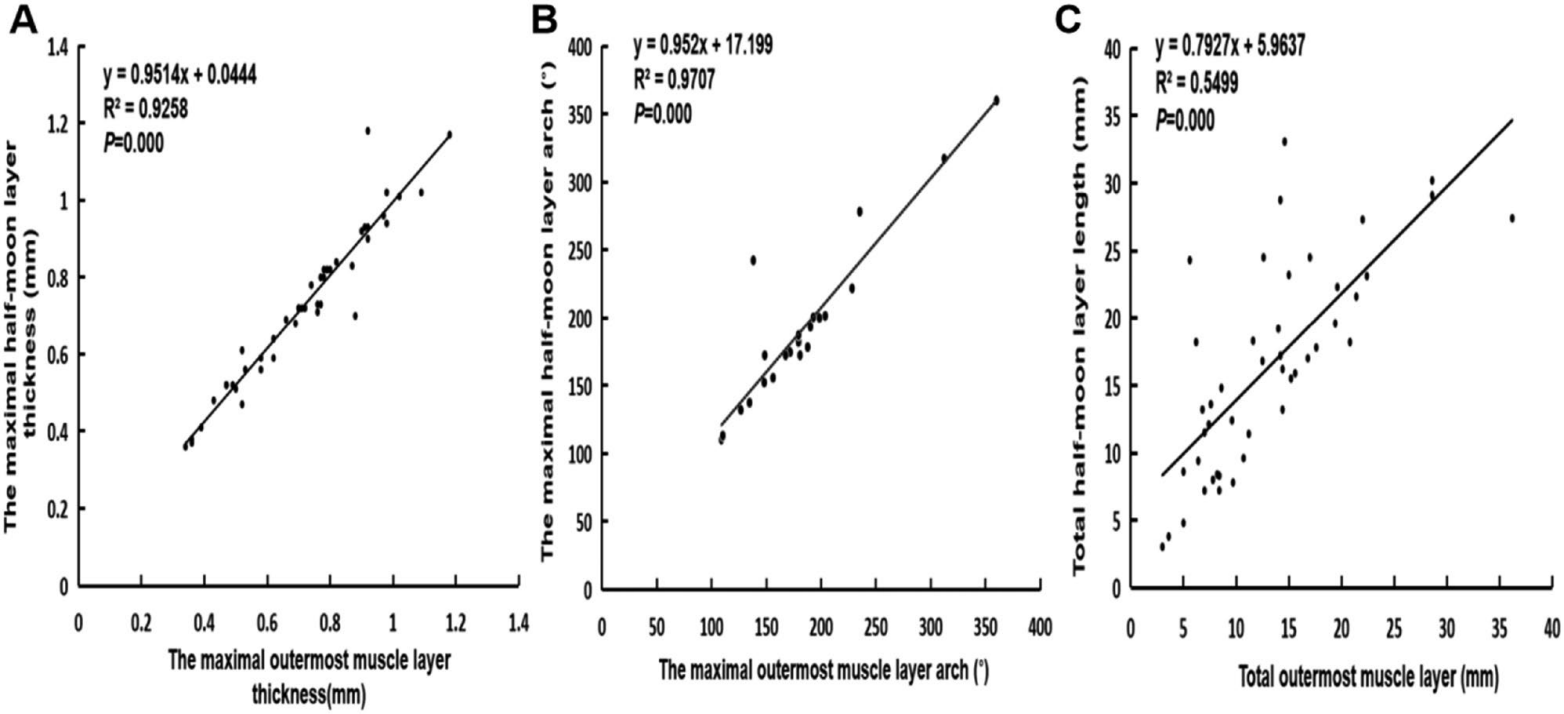
The relation of IVUS and OCT for evaluating the MB characteristics. (**A**) The relation of the maximal visible outermost muscle layer's thickness with the maximal half-moon layer's thickness. (**B**) The relation of the maximal visible outermost muscle layer's arch with the maximal half-moon layer's arch. (**C**) The relation of total visible outermost muscle layer's length with total half-moon layer's length.

Finally, we performed the correlation analysis on these 3 indexes between the half-moon layer (IVUS) and the muscle layer (OCT). Maximal thickness, arch, and total length of the half-moon layer were significantly positively related with those of the visible muscle layer, respectively (*r* = 0.962, 0.985, 0.742, *p* < 0.01) (Fig. 4).

These results revealed that a visible muscle layer around the vessel adventitia detected by the OCT could also accurately reflect the MB condition compared to the IVUS.

### Basic clinical characteristics between MB and non-MB groups detected by optical coherence tomography

Next, we retrospectively 2676 patients checked with the OCT and found that 626 patients had the phenomenon that a visible muscle layer around the vessel adventitia with the high or same density as the vessel media layer, indicated that these patients had MB in the coronary artery. Between MB and no MB groups, there was not any significant difference in gender, age, hypertension, dyslipidemia, diabetes, and diagnosis [SMI (silent myocardial ischemia), SA (stable angina), UA (unstable angina), and AMI (acute myocardial infarction)]. The MB in 626 patients detected by OCT all occurred in a left anterior descending artery (LAD), and the other 2050 patients seen by OCT had no MB. Among 202 patients who received the IVUS examination simultaneously, 45 of them had MB detected by IVUS. Of these 626 patients with MB seen by OCT, only 300 could be checked out by CAG. According to CAG, most MB (98.67%) of these patients were located in the middle segment of LAD. Finally, these patients with MB were divided into no mild MB (n = 204), moderate MB (n = 75), and severe MB (n = 21) groups using the MB classification of CAG (Table 3).

**Table 3:** Basic clinical characteristics between MB and non-MB groups detected by optical coherence tomography.

Variables	MB group (n = 626)	No MB group (n = 2050)	*p* value
Gender (male)	457 (73.00%)	1500 (73.17%)	0.959
Age (years)	62.50 ± 10.54	63.41 ± 10.61	0.063
Hypertension	421 (67.25%)	1335 (65.12%)	0.337
Dyslipidemia	388 (61.98%)	1205 (58.78%)	0.163
Diabetes	149 (23.80%)	510 (24.88%)	0.597
**Diagnosis**			0.485
SMI	44 (7.03%)	147 (7.17%)	
SA	103 (16.45%)	349 (17.02%)	
UA	401 (64.06%)	1253 (61.12%)	
AMI	78 (12.46%)	301 (14.68%)	
**Coronary artery detected OCT**			0.000
LAD	626 (100.00%)	1140 (55.61%)	
LCX	0 (0.00%)	314 (15.32%)	
RCA	0 (0.00%)	595 (29.02%)	
IVUS use	46 (7.35%)	156 (7.61%)	0.863
MB detected by IVUS	45	0 (0.00%)	0.000
**Coronary angiography**			
MB detected by CAG	300 (47.92%)	0 (0.00%)	0.000
**MB location**			0.000
Proximal LAD	0 (0.00%)	0 (0.00%)	
Middle LAD	296 (98.67%)	0 (0.00%)	
Distal LAD	4 (1.33%)	0 (0.00%)	
**MB classification**			0.000
Mild MB	204	0 (0.00%)	
Moderate MB	75	0 (0.00%)	
Severe MB	21	0 (0.00%)	

Data were expressed as n (%), mean ± SD. Variance analysis, P1; *SMI* silent myocardial ischemia, *SA* stable angina, *UA* unstable angina, *AMI* acute myocardial infarction, *LAD* left anterior descending coronary artery, *LCX* left circumflex coronary artery, *RCA* right coronary artery, *CAG* coronary angiography.

### Basic clinical and OCT characteristics among no MB, mild MB, moderate MB, and severe MB groups according to coronary angiography

According to CAG, 626 patients with MB detected by OCT were divided into no MB, mild MB, moderate MB, and severe MB groups. Among these 4 groups, there were significant differences in gender (male), age, hypertension, diabetes, and plaque formation in MB (*p* < 0.05). However, there were no notable differences in smoking, dyslipidemia, diagnosis, MB continuity, and plaque character (*p* > 0.05). 23.96% of patients had plaque formation in the MB segment. Among them, 70% of plaques were fibrotic and fatty (Table 4).

**Table 4:** Basic clinical and OCT characteristics among no MB, mild MB, moderate MB, and severe MB groups according to coronary angiography.

Variables	No MB (n = 326)	Mild MB (n = 204)	Moderate MB (n = 75)	Severe MB (n = 21)	*P*1 value	*P*2 value	*P*3 value	*P*4 value	*P*5 value	*P*6 value	*P*7 value
Gender (male)	222 (68.10%)	154 (75.49%)	65 (86.67%)	16 (76.19%)	0.008	–	–	–	–	–	–
Age (years)	63.41 ± 10.92	62.27 ± 10.05	59.40 ± 9.43	61.76 ± 10.78	0.028	0.227	0.003	0.486	0.043	0.831	0.362
Smoking	136 (41.72%)	85 (41.67%)	33 (44.00%)	14 (66.67%)	0.158	–	–	–	–	–	–
Hypertension	235 (72.09%)	132 (64.71%)	40 (53.33%)	14 (66.67%)	0.014	–	–	–	–	–	–
Dyslipidemia	202 (61.96%)	120 (58.82%)	45 (60.00%)	13 (61.90%)	0.908	–	–	–	–	–	–
Diabetes	90 (27.61%)	45 (22.06%)	13 (17.33%)	1 (4.76%)	0.031	–	–	–	–	–	–
**Diagnosis**					0.416	–	–	–	–	–	–
SMI	14 (4.38%)	7 (3.54%)	6 (8.22%)	2 (10.00%)							
SA	53 (16.56%)	33 (16.67%)	15 (20.55%)	2 (10.00%)							
UA	204 (63.75%)	137 (69.19%)	46 (63.01%)	14 (70.00%)							
AMI	49 (15.31%)	21 (10.61%)	6 (8.22%)	2 (10.00%)							
**MB continuity**					0.470	–	–	–	–	–	–
Continuity	69 (21.17%)	43 (21.08%)	21 (28.00%)	3 (14.29%)							
Discontinuity	257 (78.83%)	161 (78.92%)	54 (72.00%)	18 (85.71%)							
The maximal visible muscle layer’s thickness	0.67 ± 0.21	0.68 ± 0.19	0.76 ± 0.21	0.91 ± 0.19	0.000	0.380	0.000	0.000	0.004	0.000	0.002
The maximal visible muscle layer’s arch	224.96 ± 96.22	248.58 ± 98.33	294.64 ± 93.39	360.00 ± 0.00	0.000	0.006	0.000	0.000	0.000	0.000	0.006
Total visible muscle layer’s length	10.83 ± 6.67	12.42 ± 6.91	14.66 ± 7.35	13.71 ± 8.08	0.000	0.010	0.000	0.065	0.017	0.417	0.577
Plaque in the MB	93 (28.53%)	38 (18.63%)	15 (20.00%)	4 (19.05%)	0.049	–	–	–	–	–	–
**Plaque character**					0.082	–	–	–	–	–	–
Fibrous plaque	20 (21.51%)	12 (31.58%)	4 (26.67%)	3 (75.00%)							
Lipid plaque	2 (2.15%)	0 (0.00%)	2 (13.33%)	0 (0.00%)							
Fibrotic and calcific plaque	2 (2.15%)	0 (0.00%)	0 (0.00%)	0 (0.00%)							
Fibrotic and fatty plaque	69 (74.19%)	26 (68.42%)	9 (60.00%)	1 (25.00%)							

Data were expressed as n (%), mean ± SD. Mild MB vs. No MB, P2; Moderate vs. No MB, P3; Severe MB vs. No MB, P4; Moderate MB vs Mild MB, P5; severe MB vs Mild MB, P6; Moderate vs. Severe MB, P7.

Maximal thickness, arch, and total length of a visible muscle layer around the vessel adventitia were measured. We found that these indexes mentioned above showed significant differences among these 4 groups (*p* < 0.05). Furthermore, maximal thickness and the arch of a visible muscle layer increased with the severity of MB, but its total length was not changed like that (Table 4).

These results suggested that the more extensive the thickness and arch of a visible muscle layer around the vessel adventitia, the more severe the myocardial bridge.

### PCI strategy and the 1-year follow-up ISR outcome guided by OCT among no MB, mild MB, moderate MB, and severe MB groups according to coronary angiography

According to the current PCI guideline, there was a significant difference in the PCI procedure among the 4 groups (*p* < 0.01), but PCI strategy (including DES and DCB) showed no significant difference (*p* > 0.05). The incidence of MB not covered with DES among the 4 groups was markedly high compared to MB covered with DES (*p* < 0.05). Moreover, the incidence of MB completely covered with DES was notably low compared with MB partially covered with DES (*p* < 0.01). Under the guidance of OCT, there was no coronary perforation in these patients with MB covered with DES.

After 1-year follow-up, there was no significant difference in ISR in MB not covered with DES among the 4 groups (*p* > 0.05). Besides, ISR in MB with and without covered with DES had no significant difference among these 4 groups (*p* > 0.05). However, ISR in DES covered with MB showed a notable difference among 4 groups (*p* < 0.05), and ISR in DES aggravated with the MB severity (Table 5).

**Table 5:** PCI strategy and the 1-year follow-up ISR outcome guided by OCT among no MB, mild MB, moderate MB, and severe MB groups according to coronary angiography.

Variables	No MB (CAG, n = 326)	Mild MB (CAG, n = 204)	Moderate MB (CAG, n = 75)	Severe MB (CAG, n = 21)	Total	*p* value
PCI	269 (82.52%)	163 (79.90%)	50 (66.67%)	13 (61.90%)	495 (79.07%)	0.004
**PCI strategy**						0.401
DCB	6 (2.23%)	1 (0.61%)	0 (0.00%)	0 (0.00%)	7 (1.41%)	
DES	263 (97.77%)	162 (99.39%)	50 (100.00%)	13 (100.00%)	488 (98.59%)	
**DES location**						0.034
MB not covered with DES (OCT)	148 (56.27%)	109 (67.28%)	33 (66.00%)	11 (84.62%)	301 (61.68%)	
MB covered with DES (OCT)	115 (43.73%)	53 (32.72%)	17 (34.00%)	2 (15.38%)	187 (38.32%)	0.000
MB partially covered with DES	102 (88.70%)	52 (98.11%)	17 (100.00%)	2 (100.00%)	173 (92.51%)	
MB completely covered with DES	13 (11.30%)	1 (1.89%)	0 (0.00%)	0 (0.00%)	14 (7.49%)	
ISR	10 (3.80%)	6 (3.70%)	2 (4.00%)	1 (7.69%)	19 (3.89%)	0.914
ISR in MB covered with DES	5 (4.35%)	4 (7.54%)	2 (11.76%)	1 (50.00%)	12 (6.42%)	0.045
ISR in MB not covered with DES	5 (3.38%)	2 (1.83%)	0 (0.00%)	0 (0.00%)	7 (2.33%)	0.597

Data were expressed as n (%).

*PCI* percutaneous coronary artery, *DCB* drug-coated balloon, *DES* drug eluted stent, *ISR* in-stent restenosis.

These results revealed that it is safe and effective to guide DES covering the mild MB segment in patients with severe coronary lesions after checking with OCT.

## Discussion

In the present study, the major findings were as follows: (1) the characteristics of the "half-moon" sign showed by IVUS were almost consistent with a visible muscle layer around the vessel adventitia with the same or high density as the vessel media layer in patients with MB. (2) the detection rate of MB in OCT was significantly high compared to CAG, and LAD was the most location of MB. (3) the larger the thickness and arch of a visible muscle layer around the vessel adventitia, the more severe the MB in patients checked with OCT. (4) under the guidance of OCT, it was safe and effective to use DES to cover the mild MB in patients with severe lesion after 1-year follow-up.

MB is characterized by epicardial coronary artery tunneling through the myocardium, with the angiographic "milking" phenomenon and a "half-moon" echolucent detected by IVUS^9,10^. The IVUS hallmark of a tunneled segment of the coronary artery is a variable degree of compression that persists into diastole, with the typical finding of the "half-moon phenomenon", an echolucent area present only between the bridged coronary segment and epicardial tissue in the whole cardiac cycle^9,10^. However, the etiology of this phenomenon is not clear. OCT is a novel imaging technology based on light, which can be used to study tissues in vivo^20^. Its ultra-high resolution is about 10 µm, which can display the anatomical structure of the coronary artery and provide a high-quality coronary artery image^20^. Xu and his team have reported that the length of the MB measured by OCT appeared much longer than that measured by CAG, and the maximal extent of MB stenosis appeared significantly small with OCT compared to CAG, indicating that OCT can more clearlyobserve the morphology and intimal structure of MB than CAG ^21^. Recently, Liu and his colleagues have revealed that the coronary artery involved in MB was smaller and thinner than that of the adjacent non-MB segment^15^. However, no data to date reported what a "half-moon" echolucent in IVUS corresponds to after the OCT checking. After retrospectively analyzing 45 patients with MB after IVUS and OCT detection, we found that the characteristics of the half-moon sign in IVUS were highly related to the visible muscle layer around the vessel adventitia in OCT, which had the same or high density as the vessel media layer in patients with MB. As we knew, the sample size of patients with MB checked with IVUS and OCT was more than ever reported. It has revealed that the larger the thickness and radian of the "half-moon" like area was, the more serious it was in patients with MB after the IVUS checking^7,9,10^. Therefore, with more severe MB, the thickness and radian of the visible muscle layer around the vessel adventitia became more significant detected by OCT in our study.

Although CAG might seem a valuable modality for anatomic assessment, the sensitivity of CAG for the MB detection is low, generally estimated as about 5%, ranging from 0.5% and 12% in previous studies^9,10,12^. However, IVUS is usually considered as a more specific and invasive modality for the anatomic detection of MB^9,10^. During the whole cardiac cycle, IVUS presents as an echolucent "half-moon" area adjacent to the lumen^9,10^. In our study, the characteristics of the visible muscle layer around the vessel adventitia detected by OCT were highly related to an echolucent "half-moon" area checked by IVUS. Thus, the sensitivity of OCT for detecting MB was also significantly high compared to CAG. MB can be found in any epicardial artery, and most of them (70–98%) involve the LAD^22,23^. However, in the study, LAD was the only coronary artery with the MB segment by the OCT detection.

Small sample size, single-center data, and retrospective study might explain why the above phenomenon happened. MBs are mostly found in the middle segment of the LAD, and the result was similar to our finding. Unlike CAG, OCT could not only identify MB but also the plaque character^20^. Xu and his colleges have reported that stable fibrous plaques, but no lipid or other types of plaque, were found in the MB segment of all 12 patients under the guidance of OCT^21^. However, the sample size is too small. Besides the fibrous plaque, lipid, fibrous and calcified, and fibrous and lipid plaques were found in the MB segment of our study. Among them, the most common was the fibrous and lipid plaque. The MB segment is always free of atherosclerosis^24^. Midiri and his team have reported that employing computed tomography coronary angiography (CT-CA), 9.4% of patients presented the atherosclerotic lesion in the MB segment^24^. It is sensitive to use invasive coronary catheters such as OCT and IVUS to detect MB compared to CT-CA^9,10^. Therefore, in the present study, 24.0% of patients had an atherosclerotic lesion in the MB segment.

Stent into the MB segment could lead to a high rate of coronary artery perforation, stent restenosis, and stent fracture, etc.^25–27^. However, there are some conditions where a stent is required to wholly or partially cover the MB. For example, severe plaque burden results in serious narrowing in the MB segment, or there is no good distal landing zone for stent implantation^26,27^. In the present study, under the guidance of OCT, there was no perforation incidence in the MB segment wholly or partially covered with DES, revealing that covering the MB guided by OCT was a safe procedure. After a 1-year follow-up, the ISR incidence in MB with the DES coverage was comparable with MB without the DES coverage among no MB, mild MB, moderate MB, and severe MB groups. Additionally, the high incidence of in-stent restenosis is accompanied by the increased severity of MB. These results were indicated that it was safe and effective to use the DES wholly or partially cover the mild MB segment detected by the OCT.

## Limitations

It was a retrospective and single-center study. Furthermore, the sample size was relatively small.

## Conclusion

OCT could accurately evaluate the characteristics of a visible muscle layer around the vessel adventitia in patients with MB compared to IVUS. The MB detective rate of OCT was significantly high compared to CAG. Finally, covering the mild MB segment with DES was relatively safe and effective in patients with a severe coronary lesion.

## Data Availability

The data that support the findings of this study are available from the corresponding author upon reasonable request.

## References

[1] Kosinski A, Grzybiak M (2001). Myocardial bridges in the human heart: Morphological aspects. Folia Morphol..

[2] Soran O, Pamir G, Erol C, Kocakavak C, Sabah I (2000). The incidence and significance of myocardial bridge in a prospectively defined population of patients undergoing coronary angiography for chest pain. Tokai J. Exp. Clin. Med..

[3] Corban MT (2014). Myocardial bridging: Contemporary understanding of pathophysiology with implications for diagnostic and therapeutic strategies. J. Am. Coll. Cardiol..

[4] Forsdahl SH (2017). Myocardial bridges on coronary computed tomography angiography-correlation with intravascular ultrasound and fractional flow reserve. Circ. J..

[5] Risse M, Weiler G (1985). Coronary muscle bridge and its relations to local coronary sclerosis, regional myocardial ischemia and coronary spasm. A morphometric study. Z. Kardiol..

[6] Donkol RH, Saad Z (2013). Myocardial bridging analysis by coronary computed tomographic angiography in a Saudi population. World J. Cardiol..

[7] Murtaza G (2020). An updated review on myocardial bridging. Cardiovasc. Revascularization Med. Incl. Mol. Interv..

[8] Yamada R (2016). Functional versus anatomic assessment of myocardial bridging by intravascular ultrasound: Impact of arterial compression on proximal atherosclerotic plaque. J. Am. Heart Assoc..

[9] Rogers IS, Tremmel JA, Schnittger I (2017). Myocardial bridges: Overview of diagnosis and management. Congenit. Heart Dis..

[10] Mohlenkamp S, Hort W, Ge J, Erbel R (2002). Update on myocardial bridging. Circulation.

[11] Ge J (1994). Comparison of intravascular ultrasound and angiography in the assessment of myocardial bridging. Circulation.

[12] Karna SK, Chourasiya M, Parikh RP, Chaudhari T, Patel U (2020). Prevalence of myocardial bridge in angiographic population—A study from rural part of western India. J. Fam. Med. Prim. Care..

[13] Bose D, Philipp S (2008). Images in clinical medicine. High-resolution imaging of myocardial bridging. N. Engl. J. Med..

[14] Ye Z (2016). Fusiform appearance of myocardial bridging detected by OCT. JACC Cardiovasc. Imaging.

[15] Ye Z, Lai Y, Yao Y, Mintz GS, Liu X (2019). Optical coherence tomography and intravascular ultrasound assessment of the anatomic size and wall thickness of a muscle bridge segment. Catheter. Cardiovasc. Interv..

[16] Noble J, Bourassa MG, Petitclerc R, Dyrda I (1976). Myocardial bridging and milking effect of the left anterior descending coronary artery: Normal variant or obstruction?. Am. J. Cardiol..

[17] Chen SL (2015). Randomized comparison of FFR-guided and angiography-guided provisional stenting of true coronary bifurcation lesions: The DKCRUSH-VI trial (double kissing crush versus provisional stenting technique for treatment of coronary bifurcation lesions VI). JACC Cardiovasc. Interv..

[18] Zhang J (2018). Intravascular ultrasound versus angiography-guided drug-eluting stent implantation: The ULTIMATE trial. J. Am. Coll. Cardiol..

[19] Li X (2021). Optical coherence tomography predictors of target vessel myocardial infarction after provisional stenting in patients with coronary bifurcation disease. Catheter. Cardiovasc. Interv..

[20] Shi SY (2018). Correlation between pre-procedural plaque morphology and patterns of in-stent neointimal hyperplasia at 1-year follow-up in patients treated with new-generation drug-eluting stents: An optical coherence tomography based analysis. J. Interv. Cardiol..

[21] Cao HM, Jiang JF, Deng B, Xu JH, Xu WJ (2010). Evaluation of myocardial bridges with optical coherence tomography. J. Int. Med. Res..

[22] Lee MS, Chen CH (2015). Myocardial bridging: An up-to-date review. J. Invasive Cardiol..

[23] Nasr AY (2014). Myocardial bridge and coronary arteries: Morphological study and clinical significance. Folia Morphol..

[24] La Grutta L (2012). Atherosclerotic pattern of coronary myocardial bridging assessed with CT coronary angiography. Int. J. Cardiovasc. Imaging.

[25] Hao Z (2018). The outcome of percutaneous coronary intervention for significant atherosclerotic lesions in segment proximal to myocardial bridge at left anterior descending coronary artery. Int. Heart J..

[26] Kunamneni PB (2008). Outcome of intracoronary stenting after failed maximal medical therapy in patients with symptomatic myocardial bridge. Catheter. Cardiovasc. Interv..

[27] Ernst A, Bulum J, Separovic Hanzevacki J, Lovric Bencic M, Strozzi M (2013). Five-year angiographic and clinical follow-up of patients with drug-eluting stent implantation for symptomatic myocardial bridging in absence of coronary atherosclerotic disease. J. Invasive Cardiol..

